# Supplementary material for: Human leukocyte antigen variants associate with BNT162b2 mRNA vaccine response
Source: Commun Med (Lond). 2024 Apr 4;4:63. doi: 10.1038/s43856-024-00490-2 (PMC10995155; doi:10.1038/s43856-024-00490-2)
Supplement: Supplementary file 2 — Supplementary Information [file 43856_2024_490_MOESM2_ESM.pdf]

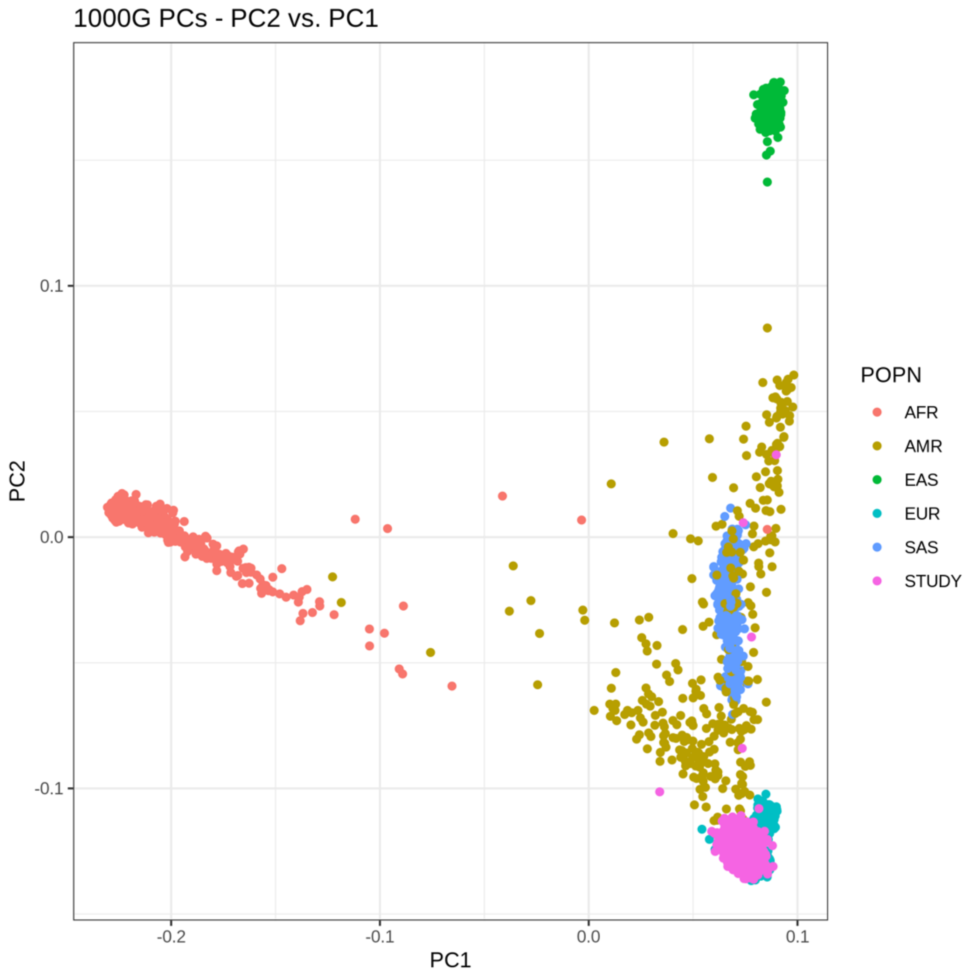

Supplementary Figure 1. PC projection of our samples into the same PC space of 1,563 subjects, from different ancestries, belonging to the 1000 Genomes Project. Pink dots represent our samples (STUDY), whereas the other colours indicate different populations (AFR, Africans, AMR, Americans, EAS, East-Asians, EUR, Europeans, SAS, South-Asians). The four pink dots not clustering with the majority of our samples (and with Europeans) were those excluded from the GWAS, since they were not of European origin.

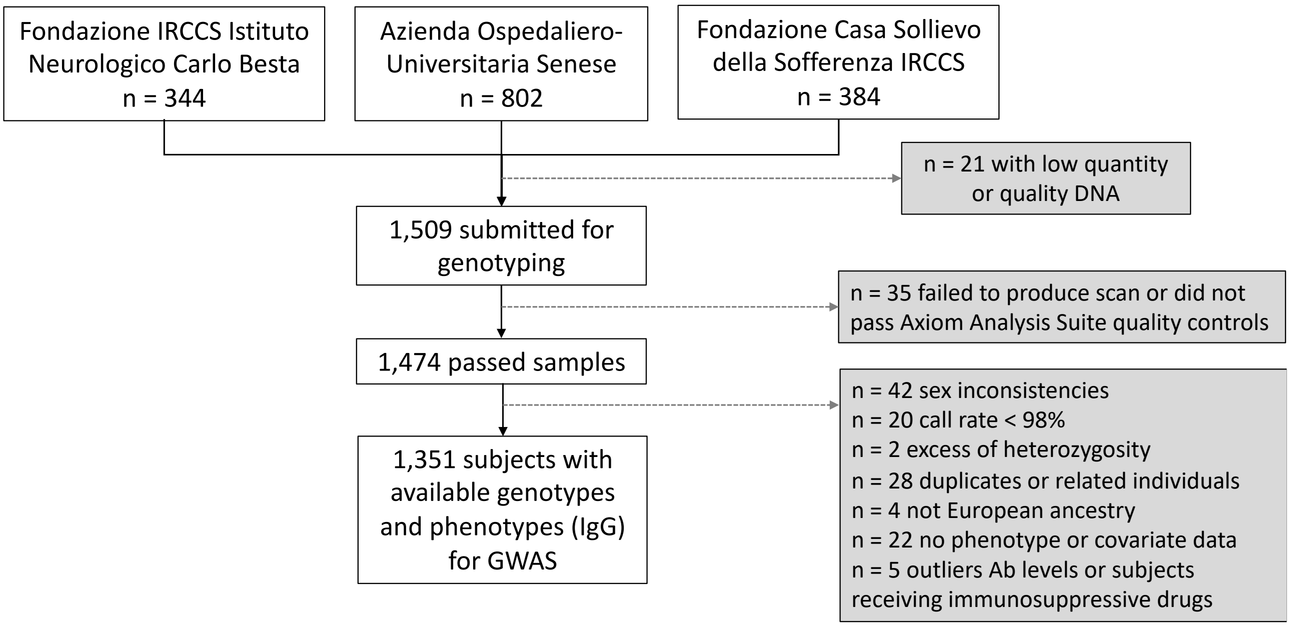

Supplementary Figure 2. Flow diagram of selected subjects for the GWAS. The final 1,351 series consisted of n=306 subjects from Milan, n=689 from Siena, and n=356 from San Giovanni Rotondo.

A.

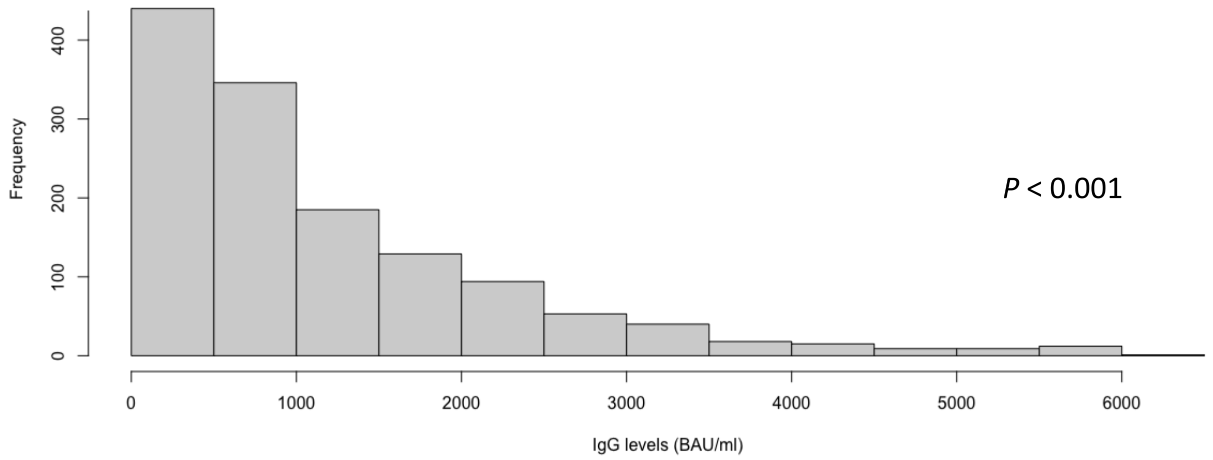

B.

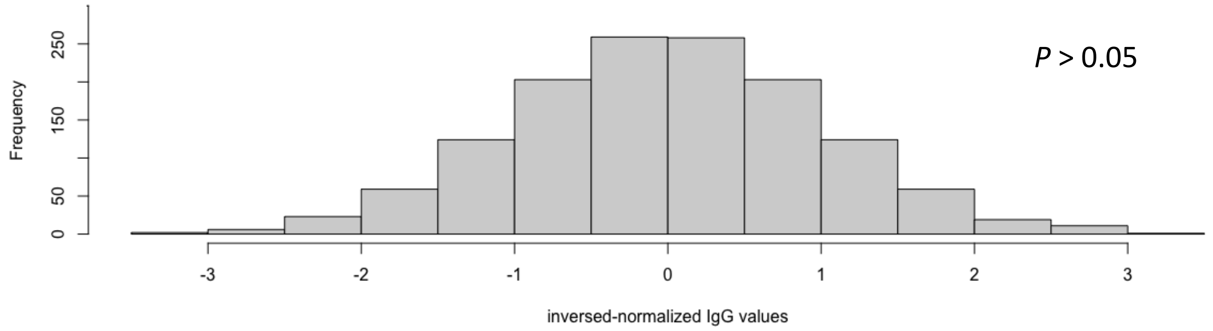

Supplementary Figure 3. Histograms representing the distribution of IgG levels in our series before (A) and after (B) inverse-normalization. *P*-values of Shapiro-Wilk test for normality are shown.

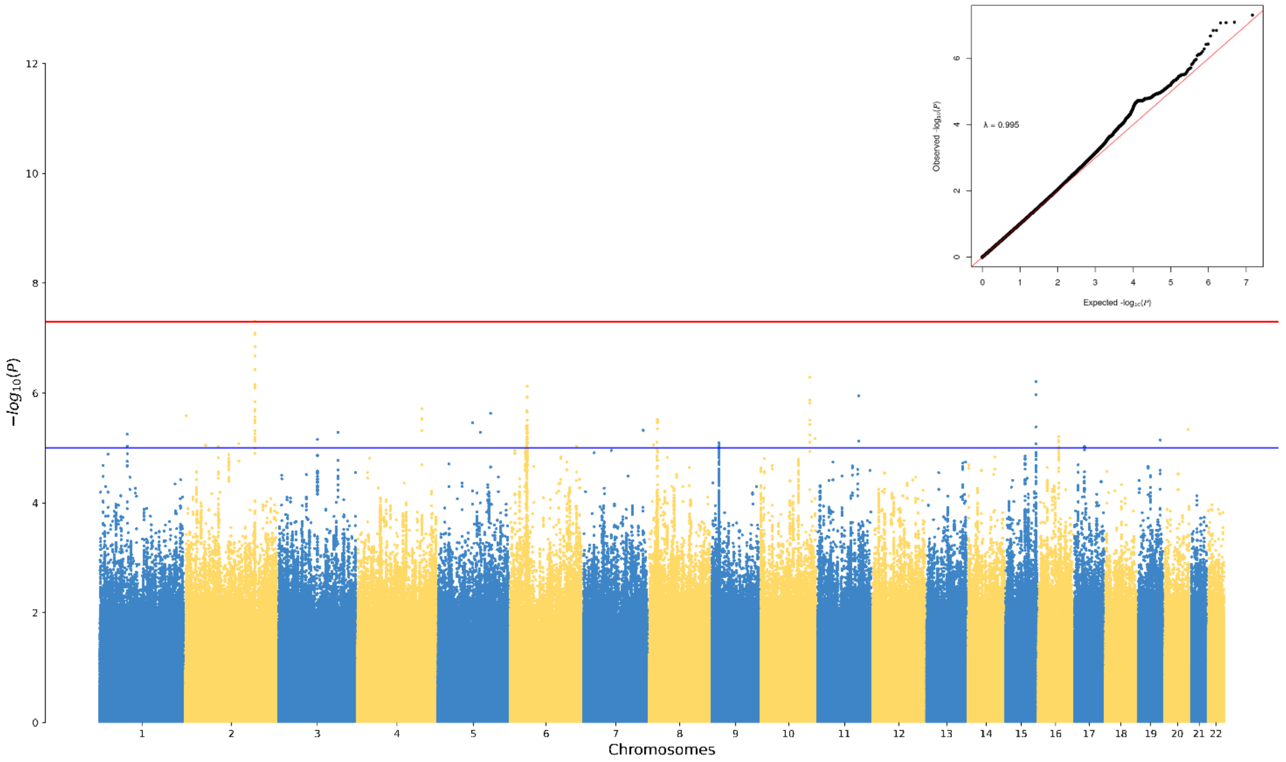

Supplementary Figure 4. **After conditioning the analysis for variants on chromosome 6 the signal on chromosome 2 (rs11692649) became statistically significant**. Manhattan plot of the results of the GWAS between anti-spike inverse-normalized IgG values and 7,339,393 imputed variants, tested in a linear regression model, using sex, age at vaccination, center, the first 5 principal components (PCs), the time interval between the second vaccine dose and the serological test, rs1632893 and rs28366135 genotypes, as covariates. SNPs are plotted on the x-axis according to their genomic position (GChr37, hg19 release), and *P*-values ($-\log_{10}P$) for their association with IgG levels on the y-axis. The horizontal red line represents the threshold of genome-wide significance (*P*-value$<5.0\text{x}10^{-8}$). In the up-right corner is shown the Q-Q plot of observed and expected *P* values. Genomic inflation factor (λ) is reported.

Supplementary Table 1. Linkage disequilibrium among the 12 top-significant HLA-alleles

D' or r^2	HLA_A*03:01	HLA_C*12:02	HLA_DQB1*06:01	HLA_DRB1*15:02	HLA_B*52:01	HLA_DQB1*02:01	HLA_DRB1*14:01	HLA_DQA1*01:01	HLA_DQA1*02:01	HLA_A*29:02	HLA_DQB1*05:03	HLA_DRB1*07:01
**HLA_A*03:01**	1,000	0,002	0,000	0,000	0,002	0,001	0,000	0,002	0,000	0,000	0,000	0,000
**HLA_C*12:02**	0,834	1,000	0,523	0,553	0,931	0,005	0,001	0,001	0,001	0,000	0,000	0,001
**HLA_DQB1*06:01**	0,007	0,729	1,000	0,947	0,530	0,005	0,001	0,002	0,003	0,000	0,001	0,003
**HLA_DRB1*15:02**	0,001	0,757	1,000	1,000	0,560	0,005	0,001	0,002	0,003	0,000	0,001	0,003
**HLA_B*52:01**	0,887	0,982	0,734	0,776	1,000	0,005	0,001	0,002	0,001	0,000	0,000	0,001
**HLA_DQB1*02:01**	0,042	1,000	1,000	1,000	1,000	1,000	0,015	0,046	0,375	0,019	0,016	0,354
**HLA_DRB1*14:01**	0,004	0,638	0,972	0,904	0,994	1,000	1,000	0,290	0,007	0,001	0,837	0,007
**HLA_DQA1*01:01**	0,060	0,394	0,677	0,715	0,710	1,000	0,928	1,000	0,028	0,004	0,321	0,027
**HLA_DQA1*02:01**	0,016	0,562	1,000	1,000	0,463	0,782	0,866	1,000	1,000	0,026	0,005	0,956
**HLA_A*29:02**	0,301	1,000	1,000	1,000	1,000	0,445	0,665	0,951	0,414	1,000	0,001	0,028
**HLA_DQB1*05:03**	0,009	0,012	0,712	0,629	0,443	1,000	0,929	0,961	0,738	0,712	1,000	0,005
**HLA_DRB1*07:01**	0,020	0,681	1,000	1,000	0,574	0,770	0,877	1,000	0,993	0,419	0,744	1,000

Supplementary Note 1. List of contributors to GEN-COVID Multicenter study

(https://sites.google.com/dbm.unisi.it/gen-covid)

Francesca Mari[1,2,3], Sergio Daga[1,2], Ilaria Meloni[1,2], Mirella Bruttini[1,2,3], Susanna Croci[1,2], Mirjam Lista[1,2], Debora Maffeo[1,2], Elena Pasquinelli[1,2], Enrica Antolini[1,2], Simona Letizia Basso[1,2], Samantha Minetto[1,2], Giulia Rollo[1,2], Angela Rina[1,2], Martina Rozza[1,2], Rossella Tita[3], Maria Antonietta Mencarelli[3], Caterina Lo Rizzo[3], Anna Maria Pinto[3], Francesca Ariani[1,2,3], Francesca Montagnani[2,4], Mario Tumbarello[2,4], Ilaria Rancan[2,4], Massimiliano Fabbiani[4], Paolo Cameli[5], David Bennett[5], Federico Anedda[6], Simona Marcantonio[6], Sabino Scolletta[6], Federico Franchi[6], Maria Antonietta Mazzei[7], Susanna Guerrini[7], Edoardo Conticini[8], Luca Cantarini[8], Bruno Frediani[8], Danilo Tacconi[9], Chiara Spertilli Raffaelli[9], Arianna Emiliozzi[9], Marco Feri[10], Alice Donati[10], Raffaele Scala[11], Luca Guidelli[11], Genni Spargi[12], Marta Corridi[12], Cesira Nencioni[13], Leonardo Croci[13], Gian Piero Caldarelli[14], Davide Romani[15], Paolo Piacentini[15], Maria Bandini[15], Elena Desanctis[15], Silvia Cappelli[15], Anna Canaccini[16], Agnese Verzuri[16], Valentina Anemoli[16], Manola Pisani[16], Agostino Ognibene[17], Maria Lorubbio[17], Alessandro Pancrazzi[17], Massimo Vaghi[18], Antonella D'Arminio Monforte[19], Federica Gaia Miraglia[19], Mario U. Mondelli[20,21], Stefania Mantovani[20], Raffaele Bruno[20,22], Marco Vecchia[20], Marcello Maffezzoni[22], Enrico Martinelli[23], Massimo Girardis[24], Stefano Busani[24], Sophie Venturelli[24], Andrea Cossarizza[25], Andrea Antinori[26], Alessandra Vergori[26], Stefano Rusconi[27,28], Matteo Siano[28], Arianna Gabrieli[28], Agostino Riva[27,28], Daniela Francisci[29], Elisabetta Schiaroli[29], Carlo Pallotto[29], Saverio Giuseppe Parisi[30], Monica Basso[30], Sandro Panese[31], Stefano Baratti[31], Pier Giorgio Scotton[32], Francesca Andretta[32], Mario Giobbia[32], Renzo Scaggiante[33], Francesca Gatti[33], Francesco Castelli[34], Eugenia Quiros-Roldan[34], Melania Degli Antoni[34], Isabella Zanella[35,36], Matteo della Monica[37], Carmelo Piscopo[37], Mario Capasso[38,39], Roberta Russo[38,39], Immacolata Andolfo[38,39], Achille Iolascon[38,39], Giuseppe Fiorentino[40], Massimo Carella[41], Marco Castori[41], Giuseppe Merla[38,42], Gabriella Maria Squeo[42], Filippo Aucella[43], Pamela Raggi[44], Rita Perna[44], Matteo Bassetti[45,46], Antonio Di Biagio[45,46], Maurizio Sanguinetti[47,48], Luca Masucci[47,48], Alessandra Guarnaccia[47], Serafina Valente[49], Alex Di Florio[49], Marco Mandalà[50], Alessia Giorli[50], Lorenzo Salerni[50], Patrizia Zucchi[51], Pierpaolo Parravicini[51], Elisabetta Menatti[52], Tullio Trotta[53], Ferdinando Giannattasio[53], Gabriella Coiro[53], Fabio Lena[54], Gianluca Lacerenza[54], Cristina Mussini[55], Luisa Tavecchia[56], Lia Crotti[57,58,59,60,61], Gianfranco Parati[57,58], Roberto Menè[57,58], Maurizio Sanarico[62], Marco Gori[63,64], Francesco Raimondi[65], Alessandra Stella[65], Filippo Biscarini[66], Tiziana Bachetti[67], Maria Teresa La Rovere[68], Maurizio Bussotti[69], Serena Ludovisi[70], Katia Capitani[71], Simona Dei[72], Sabrina Ravaglia[73], Annarita Giliberti[74], Giulia Gori[74], Rosangela Artuso[74], Elena Andreucci[74], Angelica Pagliazzi[74], Erika Fiorentini[74], Antonio Perrella[75], Francesco Bianchi[2,75], Paola Bergomi[76], Emanuele Catena[76], Riccardo Colombo[76], Sauro Luchi[77], Giovanna Morelli[77], Paola Petrocelli[77], Sarah Iacopini[77], Sara Modica[77], Silvia Baroni[78], Giulia Micheli[79], Marco Falcone[80], Donato Urso[80], Giusy Tiseo[80], Tommaso Matucci[80], Davide Grassi[81], Claudio Ferri[81], Franco Marinangeli[82], Francesco Brancati[83,84], Antonella Vincenti[85], Valentina Borgo[85], Stefania Lombardi[85], Mirco Lenzi[85], Massimo Antonio Di Pietro[86], Francesca Vichi[86], Benedetta Romanin[86], Letizia Attala[86], Cecilia Costa[86], Andrea Gabbuti[86], Alessio Bellucci[86], Marta Colaneri[22], Patrizia Casprini[87], Cristoforo Pomara[88], Massimiliano Esposito[88], Roberto Leoncini[89], Michele Cirianni[89], Lucrezia Galasso[89], Marco Antonio Bellini[90], Chiara Gabbi[91], Nicola Picchiotti[63], Simone Furini[2,92]

1. Medical Genetics, University of Siena, Siena, 53100, Italy
2. Med Biotech Hub and Competence Center, Department of Medical Biotechnologies, University of Siena, Siena, 53100, Italy
3. Genetica Medica, Azienda Ospedaliero-Universitaria Senese, Siena, 53100, Italy
4. Department of Medical Sciences, Infectious and Tropical Diseases Unit, Azienda Ospedaliera Universitaria Senese, Siena, 53100, Italy
5. Unit of Respiratory Diseases and Lung Transplantation, Department of Internal and Specialist Medicine, University of Siena, Siena, 53100, Italy
6. Dept of Emergency and Urgency, Medicine, Surgery and Neurosciences, Unit of Intensive Care Medicine, Siena University Hospital, Siena, 53100, Italy
7. Department of Medical, Surgical and Neuro Sciences and Radiological Sciences, Unit of Diagnostic Imaging, University of Siena, 53100, Italy
8. Rheumatology Unit, Department of Medicine, Surgery and Neurosciences, University of Siena, Policlinico Le Scotte, Siena 53100, Italy

9. Department of Specialized and Internal Medicine, Infectious Diseases Unit, San Donato Hospital Arezzo 52100, Italy
10. Department of Emergency, Anesthesia Unit, San Donato Hospital, Arezzo, Italy
11. Department of Specialized and Internal Medicine, Pneumology Unit and UTIP, San Donato Hospital, Arezzo, 52100, Italy
12. Department of Emergency, Anesthesia Unit, Misericordia Hospital, Grosseto, 58100 Italy
13. Department of Specialized and Internal Medicine, Infectious Diseases Unit, Misericordia Hospital, Grosseto, 58100 Italy
14. Clinical Chemical Analysis Laboratory, Misericordia Hospital, Grosseto, 58100, Italy
15. Dipartimento di Prevenzione, Azienda USL Toscana Sud Est, 53100 Italy
16. Dipartimento Tecnico-Scientifico Territoriale, Azienda USL Toscana Sud Est, 53100, Italy
17. UOC Laboratorio Analisi Chimico Cliniche, Arezzo, 52100, Italy
18. Chirurgia Vascolare, Ospedale Maggiore di Crema, 26013 Italy
19. Department of Health Sciences, Clinic of Infectious Diseases, ASST Santi Paolo e Carlo, University of Milan, Milan, 20142, Italy
20. Division of Clinical Immunology - Infectious Diseases, Department of Medicine, Fondazione IRCCS Policlinico San Matteo, Pavia, 27100, Italy
21. Department of Internal Medicine and Therapeutics, University of Pavia, 27100 Italy
22. University of Pavia, Pavia, 27100 Italy
23. Department of Respiratory Diseases, Azienda Ospedaliera di Cremona, Cremona, 26100, Italy
24. Department of Anesthesia and Intensive Care, University of Modena and Reggio Emilia, Modena, 41121, Italy
25. Department of Medical and Surgical Sciences for Children and Adults, University of Modena and Reggio Emilia, Modena, 41121, Italy
26. HIV/AIDS Department, National Institute for Infectious Diseases, IRCCS, Lazzaro Spallanzani, Rome, 00161, Italy
27. III Infectious Diseases Unit, ASST-FBF-Sacco, Milan, 20146, Italy
28. Department of Biomedical and Clinical Sciences Luigi Sacco, University of Milan, Milan, 20146, Italy
29. Infectious Diseases Clinic, "Santa Maria della Misericordia" Hospital, University of Perugia, Perugia,06100, Italy
30. Department of Molecular Medicine, University of Padova, Italy
31. Clinical Infectious Diseases, Mestre Hospital, Venezia, Italy.
32. Department of Infectious Diseases, Treviso Hospital, Local Health Unit 2 Marca Trevigiana, Treviso, Italy
33. Infectious Diseases Clinic, ULSS1, Belluno, Italy
34. Department of Infectious and Tropical Diseases, University of Brescia and ASST Spedali Civili Hospital, Brescia, Italy
35. Department of Molecular and Translational Medicine, University of Brescia, Italy;
36. Clinical Chemistry Laboratory, Cytogenetics and Molecular Genetics Section, Diagnostic Department, ASST Spedali Civili di Brescia, Italy
37. Medical Genetics and Laboratory of Medical Genetics Unit, A.O.R.N. "Antonio Cardarelli", Naples, Italy
38. Department of Molecular Medicine and Medical Biotechnology, University of Naples Federico II, Naples, Italy
39. CEINGE Biotecnologie Avanzate, Naples, Italy
40. Unit of Respiratory Physiopathology, AORN dei Colli, Monaldi Hospital, Naples, Italy
41. Division of Medical Genetics, Fondazione IRCCS Casa Sollievo della Sofferenza Hospital, San Giovanni Rotondo, Italy
42. Laboratory of Regulatory and Functional Genomics, Fondazione IRCCS Casa Sollievo della Sofferenza, Foggia, Italia
43. Department of Medical Sciences, Fondazione IRCCS Casa Sollievo della Sofferenza Hospital, San Giovanni Rotondo, Italy
44. Clinical Trial Office, Fondazione IRCCS Casa Sollievo della Sofferenza Hospital, San Giovanni Rotondo, Italy
45. Department of Health Sciences, University of Genova, Genova, Italy

46. Infectious Diseases Clinic, Policlinico San Martino Hospital, IRCCS for Cancer Research Genova, Italy
47. Microbiology, Fondazione Policlinico Universitario Agostino Gemelli IRCCS, Catholic University of Medicine, Rome, Italy
48. Department of Laboratory Sciences and Infectious Diseases, Fondazione Policlinico Universitario A. Gemelli IRCCS, Rome, Italy
49. Department of Cardiovascular Diseases, University of Siena, Siena, Italy
50. Otolaryngology Unit, University of Siena, Italy
51. Department of Internal Medicine, ASST Valtellina e Alto Lario, Sondrio, Italy
52. Study Coordinator Oncologia Medica e Ufficio Flussi Sondrio, Italy
53. First Aid Department, Luigi Curto Hospital, Polla, Salerno, Italy
54. Department of Pharmaceutical Medicine, Misericordia Hospital, Grosseto, Italy.
55. Infectious Diseases Clinics, University of Modena and Reggio Emilia, Modena, Italy
56. U.O.C. Medicina, ASST Nord Milano, Ospedale Bassini, Cinisello Balsamo (MI), Italy
57. Istituto Auxologico Italiano, IRCCS, Department of Cardiovascular, Neural and Metabolic Sciences, San Luca Hospital, Milan, Italy
58. Department of Medicine and Surgery, University of Milano-Bicocca, Milan, Italy
59. Istituto Auxologico Italiano, IRCCS, Center for Cardiac Arrhythmias of Genetic Origin, Milan, Italy
60. Istituto Auxologico Italiano, IRCCS, Laboratory of Cardiovascular Genetics, Milan, Italy
61. Member of the European Reference Network for Rare, Low Prevalence and Complex Diseases of the Heart-ERN GUARD-Heart
62. Independent Data Scientist, Milan, Italy
63. University of Siena, DIISM-SAILAB, Siena, Italy
64. Maasai, I3S CNRS, Université Côte d'Azur, France
65. Laboratorio di Biologia Bio@SNS, Scuola Normale Superiore, Pisa, Italy
66. CNR-Consiglio Nazionale delle Ricerche, Istituto di Biologia e Biotecnologia Agraria (IBBA), Milano, Italy
67. Direzione Scientifica, Istituti Clinici Scientifici Maugeri IRCCS, Pavia, Italy
68. Istituti Clinici Scientifici Maugeri IRCCS, Department of Cardiology, Institute of Montescano, Pavia, Italy
69. Istituti Clinici Scientifici Maugeri IRCCS, Department of Cardiology, Institute of Milan, Italy
70. Fondazione IRCCS Ca' Granda Ospedale Maggiore Policlinico, Milan, Italy
71. Core Research Laboratory, ISPRO, Florence, Italy
72. Health Management, Azienda USL Toscana Sud Est, Tuscany, Italy
73. IRCCS C. Mondino Foundation, Pavia, Italy
74. Medical Genetics Unit, Meyer Children's University Hospital, Firenze, Italy
75. Department of Medicine, Pneumology Unit, Misericordia Hospital, Grosseto, Italy.
76. Department of Anesthesia and Intensive Care Unit, ASST Fatebenefratelli Sacco, Luigi Sacco Hospital, Polo Universitario, University of Milan, Milan, Italy
77. Infectious Disease Unit, Hospital of Lucca, Italy
78. Department of Diagnostic and Laboratory Medicine, Institute of Biochemistry and Clinical Biochemistry, Fondazione Policlinico Universitario A. Gemelli IRCCS, Catholic University of the Sacred Heart, Rome, Italy.
79. Clinic of Infectious Diseases, Catholic University of the Sacred Heart, Rome, Italy
80. Department of Clinical and Experimental Medicine, Infectious Diseases Unit, University of Pisa, Pisa, Italy
81. Department of Clinical Medicine, Public Health, Life and Environment Sciences, University of L'Aquila, Italy
82. Anesthesiology and Intensive Care, University of L'Aquila, L'Aquila, Italy
83. Department of Life, Health and Environmental Sciences, University of L'Aquila, 67100, L'Aquila, Italy
84. Human Functional Genomics Laboratory, IRCCS San Raffaele Roma, 00167, Rome, Italy
85. Infectious Disease Unit, Hospital of Massa, Italy
86. Infectious Diseases Unit, Santa Maria Annunziata Hospital, USL Centro, Florence, Italy
87. Laboratory of Clinical Pathology and Immunoallergy, Florence-Prato, Italy

88. Department of Medical, Surgical and Advanced Technologies "G.F. Ingrassia", University of Catania, Catania, Italy
89. Laboratorio Patologia Clinica, Azienda Ospedaliero-Universitaria Senese, Siena, Italy
90. Ambulatory Chronic Polipathology of Siena, Department of Medicine, Surgery and Neurosciences, University of Siena, Siena, Italy
91. Department of Biosciences and Nutrition, Karolinska Institutet, Stockholm, Sweden
92. Bioinformatics, University of Bologna, Italy
