# Supplementary material for: Human leukocyte antigen variants associate with BNT162b2 mRNA vaccine response
Source: Commun Med (Lond). 2024 Apr 4;4:63. doi: 10.1038/s43856-024-00490-2 (PMC10995155; doi:10.1038/s43856-024-00490-2)
Supplement: Supplementary file 3 — Description of Additional Supplementary Files [file 43856_2024_490_MOESM3_ESM.pdf]

Description of Additional Supplementary Files

File Name: Supplementary Data 1

Description: Variants significantly (P-value$<5.0\text{x}10^{-8}$) associated with IgG levels after vaccination and their linkage disequilibrium values with the top-significant variant.

File Name: Supplementary Data 2

Description: Summary statistics of the HLA locus analysis.

File Name: Supplementary Data 3

Description: Numerical data underlying Figure 2. (A) Summary statistics; (B) linkage disequilibrium data.

File Name: Supplementary Data 4

Description: Numerical data underlying Figure 3. (A) Summary statistics underlying Figure 3A; (B) linkage disequilibrium data underlying Figure 3A; (C) Summary statistics underlying Figure 3B; (D) linkage disequilibrium data underlying Figure 3B.
