# Supplementary material for: Human leukocyte antigen variants associate with BNT162b2 mRNA vaccine response
Source: Commun Med (Lond). 2024 Apr 4;4:63. doi: 10.1038/s43856-024-00490-2 (PMC10995155; doi:10.1038/s43856-024-00490-2)
Supplement: Supplementary file 8 — Reporting summary [file 43856_2024_490_MOESM8_ESM.pdf]

nature portfolio

Corresponding author(s): Francesca Colombo

Last updated by author(s): Jan 10, 2024

Reporting Summary

Nature Portfolio wishes to improve the reproducibility of the work that we publish. This form provides structure for consistency and transparency in reporting. For further information on Nature Portfolio policies, see our Editorial Policies and the Editorial Policy Checklist.

Statistics

For all statistical analyses, confirm that the following items are present in the figure legend, table legend, main text, or Methods section.

n/a	Confirmed	
☐	☒	The exact sample size (*n*) for each experimental group/condition, given as a discrete number and unit of measurement
☐	☒	A statement on whether measurements were taken from distinct samples or whether the same sample was measured repeatedly
☐	☒	The statistical test(s) used AND whether they are one- or two-sided<br>*Only common tests should be described solely by name; describe more complex techniques in the Methods section.*
☐	☒	A description of all covariates tested
☐	☒	A description of any assumptions or corrections, such as tests of normality and adjustment for multiple comparisons
☐	☒	A full description of the statistical parameters including central tendency (e.g. means) or other basic estimates (e.g. regression coefficient) AND variation (e.g. standard deviation) or associated estimates of uncertainty (e.g. confidence intervals)
☐	☒	For null hypothesis testing, the test statistic (e.g. *F*, *t*, *r*) with confidence intervals, effect sizes, degrees of freedom and *P* value noted<br>*Give P values as exact values whenever suitable.*
☒	☐	For Bayesian analysis, information on the choice of priors and Markov chain Monte Carlo settings
☒	☐	For hierarchical and complex designs, identification of the appropriate level for tests and full reporting of outcomes
☐	☒	Estimates of effect sizes (e.g. Cohen's *d*, Pearson's *r*), indicating how they were calculated

Our web collection on statistics for biologists contains articles on many of the points above.

Software and code

Policy information about availability of computer code

Data collection	No custom softwares or algorithms were used.<br>REDCap 13.4.13 - © 2023 Vanderbilt University
Data analysis	No custom softwares or algorithm were used.<br>Axiom Analysis Suite v. 5.2.0.65 - © Themo Fisher Scientific<br>PLINK (https://www.cog-genomics.org/plink/2.0/)<br>Genotype Imputation HLA (Minimac4) 1.7.3 (https://imputationserver.sph.umich.edu/index.html#!run/imputationserver-hla)<br>HLA association and fine mapping (https://github.com/immunogenomics/HLA_analyses_tutorial)<br>R version 4.2.2

For manuscripts utilizing custom algorithms or software that are central to the research but not yet described in published literature, software must be made available to editors and reviewers. We strongly encourage code deposition in a community repository (e.g. GitHub). See the Nature Portfolio guidelines for submitting code & software for further information.

Data

Policy information about availability of data

All manuscripts must include a data availability statement. This statement should provide the following information, where applicable:

- Accession codes, unique identifiers, or web links for publicly available datasets
- A description of any restrictions on data availability
- For clinical datasets or third party data, please ensure that the statement adheres to our policy

Data are not openly available to preserve individuals' privacy under the European General Data Protection Regulation. They are available from the corresponding author upon reasonable request. Summary statistics of the GWAS are available in the GWAS catalog (accession n. GCST90305767)

Research involving human participants, their data, or biological material

Policy information about studies with human participants or human data. See also policy information about sex, gender (identity/presentation), and sexual orientation and race, ethnicity and racism.

Reporting on sex and gender	In our study we used the biological attribute of sex as possible confounding factor in the genetic analyses. Sex attribute derived from genetic data and only data matching self-reported sex information were included in the analyses.
Reporting on race, ethnicity, or other socially relevant groupings	The studies did not use any constructs of race and/or ethnicity.<br>Principal component analysis of genotype data was used to determine genetic ancestry of enrolled individuals. The first five principal components were used as covariates in the linear regression model with genotypes in order to account for possible population stratification an substructures.
Population characteristics	The participants were primarily hospital workers at the three Italian recruiting hospitals (Fondazione IRCCS Istituto Neurologico Carlo Besta in Milan, Azienda Ospedaliero-Universitaria Senese in Siena, and Fondazione Casa Sollievo della Sofferenza, IRCCS, San Giovanni Rotondo), who received two doses of the BNT162b2 anti-COVID-19 vaccine. They all signed written informed consent to participate to the study. The majority of subjects were females and the median age at vaccination was 48 years. The median time interval between vaccination and IgG measurement was 40 days.
Recruitment	Recruitment was done among vaccinated workers of the three recuiting hospitals (Fondazione IRCCS Istituto Neurologico Carlo Besta in Milan, Azienda Ospedaliero-Universitaria Senese in Siena, and Fondazione Casa Sollievo della Sofferenza, IRCCS, San Giovanni Rotondo), in the period spanned from December 27th 2020 and May 15th 2021.
Ethics oversight	University Hospital (Azienda ospedaliero-universitaria Senese) ethical review board, Siena, Italy.<br>Ethics Committee of IRCCS Istituto Tumori "Giovanni Paolo II", Bari at Fondazione Casa Sollievo della Sofferenza, San Giovanni Rotondo (FG), Italy.<br>Ethics Committee Regione Lombardia, Sezione Fondazione IRCCS Istituto Neurologico Carlo Besta, Milan, Italy.

Note that full information on the approval of the study protocol must also be provided in the manuscript.

Field-specific reporting

Please select the one below that is the best fit for your research. If you are not sure, read the appropriate sections before making your selection.

☒ Life sciences ☐ Behavioural & social sciences ☐ Ecological, evolutionary & environmental sciences

For a reference copy of the document with all sections, see nature.com/documents/nr-reporting-summary-flat.pdf

Life sciences study design

All studies must disclose on these points even when the disclosure is negative.

Sample size	For this study we did not calculated the sample size, that was determined by the availablity of samples from anti-COVID-19 vaccinated subjects.
Data exclusions	Genotype and phenotype data quality control steps led to the exclusion of samples with low genotyping call rate, high heterozygosity, inconsistencies between self-reported and genetic sex, duplicated and related samples (to the third degree of relatedness). Four non-European individulas were not included in the analysis. Also, subjects with no full covariate data available were excluded, as well as, individuals with outliers levels of IgG or treated with immunosuppressive drugs.
Replication	Our study could be considered a replication of that by Mentzer AJ et al. (Nat. Med. 2023), in a completely independent series. We independently confirmed and extended their result of an association between HLA locus and the antibody levels produced in response to vaccination. Some differences in the top significant SNPs and alleles between the two studies were observed.
Randomization	No randomization was done in our study. All hospital workers received the same type of vaccine in Italy in the period of recruitment.
Blinding	Blinding was not relevant to our study. All participants were vaccinated with the same vaccine.

Reporting for specific materials, systems and methods

We require information from authors about some types of materials, experimental systems and methods used in many studies. Here, indicate whether each material, system or method listed is relevant to your study. If you are not sure if a list item applies to your research, read the appropriate section before selecting a response.

Materials & experimental systems

n/a	Involved in the study
☒	☐ Antibodies
☒	☐ Eukaryotic cell lines
☒	☐ Palaeontology and archaeology
☒	☐ Animals and other organisms
☒	☐ Clinical data
☒	☐ Dual use research of concern
☒	☐ Plants

Methods

n/a	Involved in the study
☒	☐ ChIP-seq
☒	☐ Flow cytometry
☒	☐ MRI-based neuroimaging

Plants

Seed stocks

Report on the source of all seed stocks or other plant material used. If applicable, state the seed stock centre and catalogue number. If plant specimens were collected from the field, describe the collection location, date and sampling procedures.

Novel plant genotypes

Describe the methods by which all novel plant genotypes were produced. This includes those generated by transgenic approaches, gene editing, chemical/radiation-based mutagenesis and hybridization. For transgenic lines, describe the transformation method, the number of independent lines analyzed and the generation upon which experiments were performed. For gene-edited lines, describe the editor used, the endogenous sequence targeted for editing, the targeting guide RNA sequence (if applicable) and how the editor was applied.

Authentication

Describe any authentication procedures for each seed stock used or novel genotype generated. Describe any experiments used to assess the effect of a mutation and, where applicable, how potential secondary effects (e.g. second site T-DNA insertions, mosiacism, off-target gene editing) were examined.
